# Post-traumatic flexion contractures of the elbow: Operative treatment via the limited lateral approach

**DOI:** 10.1186/1749-799X-3-39

**Published:** 2008-09-10

**Authors:** Mark D Brinsden, Andrew J Carr, Jonathan L Rees

**Affiliations:** 1 The Nuffield Department of Orthopaedic Surgery, University of Oxford, Oxford, UK

## Abstract

Varying surgical techniques, patient groups and results have been described regards the surgical treatment of post traumatic flexion contracture of the elbow. We present our experience using the limited lateral approach on patients with carefully defined contracture types.

Surgical release of post-traumatic flexion contracture of the elbow was performed in 23 patients via a limited lateral approach. All patients had an established flexion contracture with significant functional deficit. Contracture types were classified as either extrinsic if the contracture was not associated with damage to the joint surface or as intrinsic if it was.

Overall, the mean pre-operative deformity was 55 degrees (95%CI 48 – 61) which was corrected at the time of surgery to 17 degrees (95%CI 12 – 22). At short-term follow-up (7.5 months) the mean residual deformity was 25 degrees (95%CI 19 – 30) and at medium-term follow-up (43 months) it was 32 degrees (95%CI 25 – 39). This deformity correction was significant (p < 0.01). One patient suffered a post-operative complication with transient dysaesthesia in the distribution of the ulnar nerve, which had resolved at six weeks. Sixteen patients had an extrinsic contracture and seven an intrinsic. Although all patients were satisfied with the results of their surgery, patients with an extrinsic contracture had significantly (p = 0.02) better results than those with an intrinsic contracture. (28 degrees compared to 48 degrees at medium term follow up).

Surgical release of post-traumatic flexion contracture of the elbow via a limited lateral approach is a safe technique, which reliably improves extension especially for extrinsic contractures. In this series all patients with an extrinsic contracture regained a functional range of movement and were satisfied with their surgery.

## Introduction

Elbow Stiffness with loss of function is a common disabling problem that usually arises as a complication of trauma [1-5], but may also occur following burns[6,7]. or head injury [8,9] or in association with degenerative, inflammatory or haemophiliac [10] arthropathy and congenital malformations [11]. The degree of stiffness is related to the severity of the injury and the duration of immobilisation at initial treatment [12,13]. Loss of elbow extension commonly produces a significant functional deficit [14]. Elbow contractures can be classified as extrinsic or intrinsic according to the underlying aetiology [15]. Extrinsic contractures involve the peri-articular soft-tissues with a normal or near normal articular surface. Intrinsic factors include disruption of the normal articular surface, osteophytes, intra-articular loose bodies and secondary osteoarthritis.

When non-operative treatments such as static or dynamic splinting [16-22] fail then surgery is often considered. Many surgical techniques have been described for established contractures with significant functional impairment. These include: manipulation-under-anaesthesia [23]; arthroscopic release [24-26]; open capsulectomy via anterior [27-31], posterior [13], medial [32,33], lateral [30,34-37], or combined approaches [38].

We present our experience of the 'mini-open' lateral approach to the elbow to correct an extension deficit in a series of patients with an established post-traumatic flexion contracture of both intrinsic and extrinsic types [35]. This approach facilitates access to the anterior capsule, the lateral ligament complex and radio-capitellum joint. It is also possible to access the posterior part of the elbow joint and olecranon of required.

## Methods

**Figure 1:**
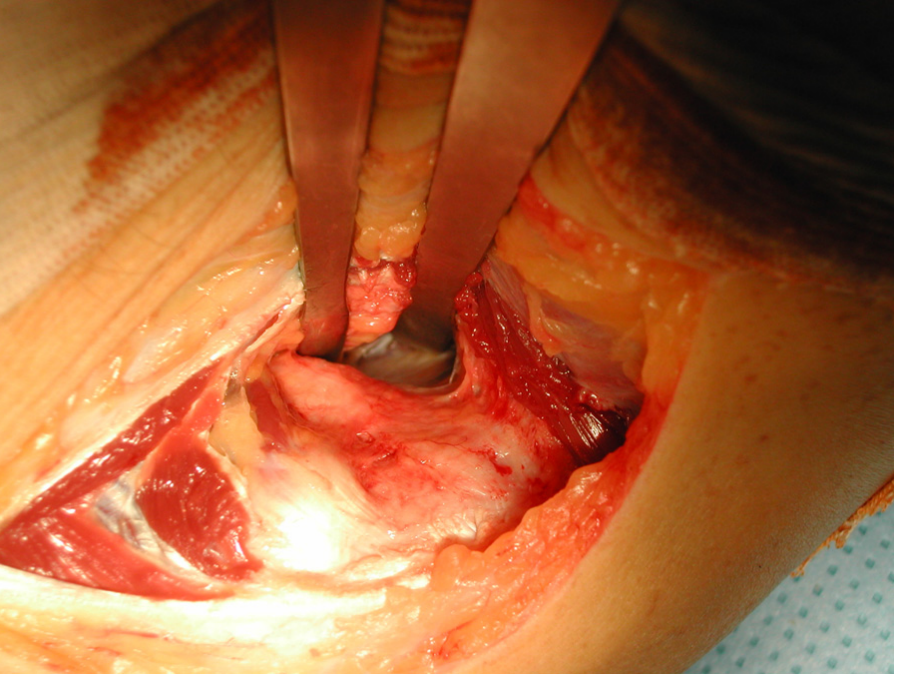
A clinical photograph showing the anterior capsule of the elbow through the lateral approach.

**Figure 2:**
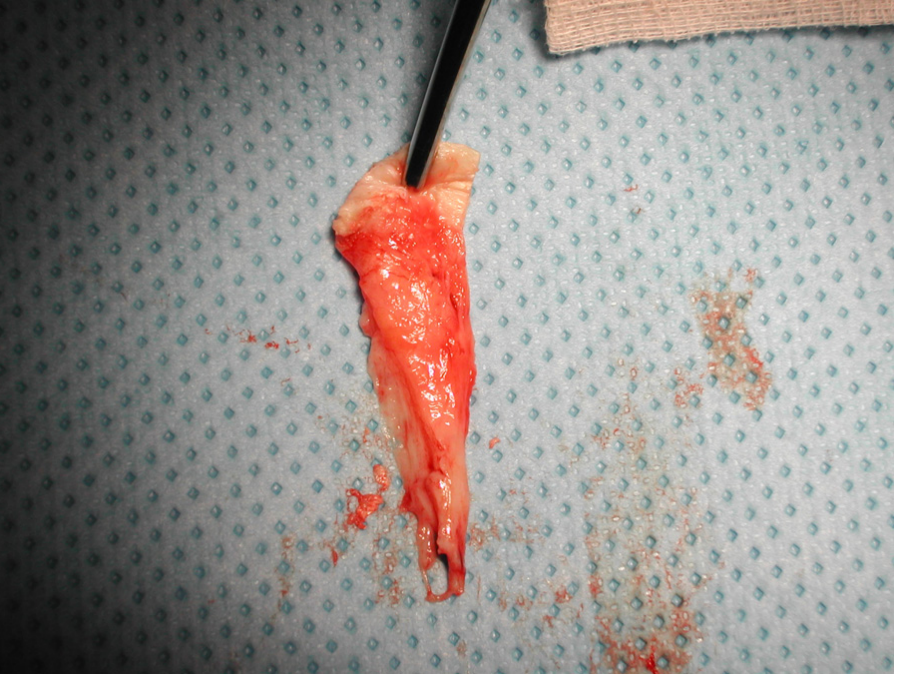
A clinical photograph showing the excised anterior capsule.

Between 1998 and 2004, 23 patients referred to our unit were treated surgically for a post traumatic flexion contracture of the elbow. The indication for surgery in all was an established functionally significant extension deficit that had failed non-operative treatment with at least 9 months having elapsed since injury. In each case the contracture was classified as extrinsic or intrinsic after assessment with clinical examination and plain radiographs and the pre-operative flexion contracture recorded. All patients consented to have their surgery under general anaesthesia and regional block with a tourniquet. The lateral column approach was used with a small 8 cm (10 cm if larger patient) incision centred over the lateral epicondyle (Figure 1). The same operative sequence was followed for all patients. All patients had a section of anterior capsule, extending across the entire anterior aspect of the joint, excised under direct vision (Figure 2). If the radial head was significantly damaged and determined at this point to be a block to extension then it was excised. Next if extension was still limited and the lateral collateral ligament complex appeared tight it was z-lengthened rather than sacrificed. Cases of intrinsic contracture also had any intra-articular lesion addressed. Any implanted metalwork that was easily accessible and may influence movement or cause pain was also removed as were any olecranonosteophytes identified on pre-operative imaging. If ulnar nerve symptoms and signs were present then an ulnar nerve release with subcutaneous transposition was performed via a separate medial incision. No distracting devices were used. The tourniquet was released, haemostasis secured with electro-cautery and a drain placed in the peri-articular soft-tissues. The residual "on-table" passive deformity was assessed after wound closure and before the application of dressings.

Post-operatively the limb was immobilised overnight in maximum extension using a plaster slab. The drain was removed and the cast replaced by a static, extension thermoplastic splint the next day. All patients were discharged on the first post-operative day. No prophylaxis was given to prevent heterotopic bone formation. The splint was worn continuously for two weeks and then at night for six weeks. Physiotherapy with active extension exercises commenced after two weeks in the presence of satisfactory wound healing. Short-term results were assessed by clinical review while medium-term follow-up was conducted using a telephone questionnaire and patient based deformity outlines as previously used by Morrey [39]. The telephone questionnaire consisted of two questions; 'Are you happy with the results of your surgery?' and 'In retrospect would you have the surgery again?'. These assessment methods were used as most patients were tertiary referrals to our unit, living many miles away and were reluctant to return for a further appointment as they were satisfied and doing well.

## Results

In the study group there were 15 males and 8 females. The median age was 35 yrs (range 16 – 52 yrs). The contracture was post-traumatic in all cases (fracture with dislocation n = 9; fracture n = 9; dislocation n = 3; and soft-tissue injury n = 2). Sixteen patients had an extrinsic contracture and 7 patients had an intrinsic aetiology.

All patients underwent anterior capsulectomy and additional procedures included: Z-lengthening of lateral collateral ligament n = 8; excision of radial head n = 3; removal of metalwork n = 3; excision of olecranon osteophyte n = 2; and ulna nerve transposition (via a separate medial incision) n = 2. Patient demographics, operative procedures and serial elbow deformities are listed in Table 1.

**Table 1:** Demographics of patients who underwent surgical correction of post-traumatic flexion contracture of the elbow

					Deformity (degrees)			
					
Patient	Age	Diagnosis	Classification	Operation	Pre-op	Peri-op	Short Term	Medium Term
1	30	Soft Tissue Injury	Extrinsic	AC	40	0	20	35
2	44	Dislocation	Extrinsic	AC	40	0	10	10
3	16	Fracture/Dislocation	Extrinsic	AC	60	5	15	15
4	38	Fracture	Extrinsic	AC	65	30	30	30
5	29	Fracture/Dislocation	Extrinsic	AC, ZLCL	55	30	30	40
6	48	Dislocation	Extrinsic	AC, ZLCL	60	20	30	30
7	31	Fracture	Extrinsic	AC	40	5	10	20
8	49	Dislocation	Extrinsic	AC	70	0	30	30
9	29	Fracture	Extrinsic	AC, ZLCL	45	20	20	30
10	41	Fracture/Dislocation	Extrinsic	AC	60	15	20	30
11	35	Fracture/Dislocation	Extrinsic	AC	60	10	15	40
12	16	Fracture	Extrinsic	AC	50	20	20	30
13	26	Soft Tissue Injury	Extrinsic	AC, ZLCL	70	10	10	N/A
14	52	Fracture/Dislocation	Extrinsic	AC	40	10	20	20
15	40	Fracture/Dislocation	Intrinsic	AC, ZLCL, EOO	60	20	40	40
16	29	Fracture/Dislocation	Extrinsic	AC, ZLCL	50	30	30	45
17	18	Fracture	Intrinsic	AC, ERH	70	30	40	45
18	37	Fracture/Dislocation	Intrinsic	AC, ERH	20	0	0	N/A
19	41	Fracture/Dislocation	Intrinsic	AC, EOO	60	20	40	N/A
20	26	Fracture	Extrinsic	AC, ROM	50	30	30	10
21	50	Fracture	Intrinsic	AC, ZLCL, ROM, UNT	60	40	50	40
22	43	Fracture	Intrinsic	AC, ERH	50	30	30	45
23	32	Fracture	Intrinsic	AC, ZLCL, ROM, UNT	90	30	40	70

Key: AC = Anterior Capsulectomy; ZLCL = Z-lengthening Lateral Collateral Ligament; ERH = Excision of Radial Head; EOO = Excision of OlecranonOsteophyte; ROM = Removal of Metalwork; UNT = Ulna Nerve Transposition.

Short term follow-up was available at 7.5 months (95%CI 4 – 11) in all patients and medium term follow-up at 43 months (95%CI 30 – 56) in 20 patients (87%). Overall, the mean pre-operative flexion deformity was 55 degrees (95%CI 48 – 61). Surgery reduced the mean "on-table" deformity to 17 degrees (95%CI 12 – 22). The short term mean residual deformity was 25 degrees (95%CI 19 – 30) and 32 degrees (95%CI 25 – 39) at medium term follow-up. The improvement in the fixed-flexion deformity was significant at both short-term and medium-term follow-up (paired t-test – p < 0.01).

Sub group analysis of extrinsic and intrinsic groups revealed:

**Group One (extrinsic) **patients had a mean pre-operative flexion deformity of 53 degrees (95%CI 47 – 59); a mean "on-table" correction to 13 degrees (95%CI 7 – 19); short term deformity of 20 degrees (95%CI 16 – 25); and medium term deformity of 28 degrees (95%CI 22 – 34).

**Group Two (intrinsic) **patients had a mean pre-operative flexion deformity of 57 degrees (95%CI 40 – 74); a mean "on-table" correction to 25 degrees (95%CI 15 – 35); short term deformity of 33 degrees (95%CI 21 – 46); and medium term deformity of 48 degrees (95%CI 32 – 64). The difference between the groups was significant at short term (two sample independent t-test – p = 0.02) and medium term (p = 0.05) follow-up.

All patients were satisfied with their surgery and would undergo it again. No patients reported a loss or change in their maximum flexion. One patient had a post-operative complication with transient dysaesthesia in the distribution of the ulnar nerve that lasted for six weeks. There were no cases of haematoma, infection or post-operative instability.

## Discussion

Historically, open release was performed via extensive surgical approaches such as the anterior approach that also included a biceps tenotomy [28,31]. Urbaniak used the anterior approach to perform a capsulectomy [40], but this does not allow access to the posterior structures of the elbow and is therefore not as useful. The medial approach does permit access to the anterior and posterior parts of the joint and exposes the ulnar nerve [32] but the radial head and lateral ligament complex are beyond its reach. Contracture release via the lateral approach exposes all the relevant pathology [30] and in patients with an isolated extension deficit can be performed through a "mini" lateral incision [35].

Whatever the approach, the goal of surgical treatment is to restore a functional range of movement. Morrey showed that a flexion contracture of greater than 30° has a significant effect on elbow function [14] and Kraushaar proposed that patients participating in gymnastics, racquet or throwing sports were even less tolerant of an extension deficit [41]. In our series, all but one of the patients had a pre-operative flexion contracture greater than 30° and complained of functional restriction with daily activities. The patient with a deformity of 20° felt that her functional requirements were such that this represented a significant limitation.

We used deformity outlines for medium term follow up as most patients were tertiary referrals to our unit, living many miles away and were reluctant to return for a further appointment to report a favourable outcome. Patients were asked to get a family member draw around the affected upper limb with the elbow in maximum extension and the forearm in neutral rotation, Morrey has successfully reported on this previously [39].

While the ability of surgery to restore a functional range of movement is documented in a number of studies results have been variable. Morrey [36] and Wada [32] managed to restore a functional arc in 50%, while Schindler [42] only achieved this in 30% of cases. The patients in our study did not have significant restriction of flexion and were therefore only treated for lack of extension. In 18 of the 23 cases (79%) the flexion contracture was corrected to less than 30° providing a functional range. In the sub-group of patients with extrinsic contracture all patients had a correction to less than 30°.

There remains some controversy regarding the optimal post-operative regimen following surgery. Continuous passive motion (CPM) has been advocated as an adjunct to surgery [27,30]. Morrey initially used a regimen of CPM followed by dynamic splinting [15]. This programme required a protracted in-patient stay and has been subsequently revised to three days of CPM as an in-patient followed by dynamic splinting upon discharge [12]. Wada, in a non-randomised trial, found no difference in the outcome of patients receiving CPM after surgery [32], a finding corroborated by Chantelot who reviewed the factors influencing surgery for elbow contracture [43]. In our series, the patients were splinted in maximum extension at the end of surgery. A thermoplastic moulded splint was custom-made and the patients were discharged on the first post-operative day. The splint remained in place for two weeks, after which they progressed to physiotherapy and night splinting for six weeks. Despite having a comprehensive post-operative regimen in place, the final correction at last clinical review was, on average, 5–10° less than that achieved at the time of surgery with further deterioration in the medium-term. Similar deterioration has been observed in other series [43-45], and patients need to be warned that final deformity correction is likely to fall short of that achieved at the time of surgery and discharge. Despite this all patients in our series were satisfied with their outcome.

The ulnar nerve is at risk during retraction and with one patient having a transientulnar nerve palsy, we recommend careful positioning of retractors during this procedure.

We agree with others that all pathology pertinent to this type of flexion contracture can be addressed via the limited lateral approach. We also found that patients recovered quickly with an attendant short in-patient stay (<24 hours). While careful consideration of the potential outcome should be given when using this technique for intrinsic contractures, our results show that for extrinsic contractures with an extension deficit, the limited lateral approach provides a safe reliable way of restoring a functional range in a high percentage of patients.

## Competing interests

The authors declare that they have no competing interests.

## Authors' contributions

MDB collected data, analysed results and aided manuscript writing. AJC collected data and aided manuscript writing. JLR wrote the paper. All authors read and approved the final manuscript.
